# Design, Synthesis and Antifungal Activity of Coumarin Ring-Opening Derivatives

**DOI:** 10.3390/molecules21101387

**Published:** 2016-10-17

**Authors:** Ming-Zhi Zhang, Yu Zhang, Jia-Qun Wang, Wei-Hua Zhang

**Affiliations:** Jiangsu Key Laboratory of Pesticide Science, College of Sciences, Nanjing Agricultural University, Nanjing 210095, China; mzzhang@njau.edu.cn (M.-Z.Z.); 2014811023@njau.edu.cn (Y.Z.); 2014111011@njau.edu.cn (J.-Q.W.)

**Keywords:** coumarin, strobilurin, synthesis, ring-opening reaction, antifungal activity

## Abstract

Based on our initial design, we synthesized two series of coumarin ring-opening derivatives by the reactions of hydrolysis and methylation. Results of antifungal screening in vitro showed that the target compounds exhibited potent activity against the six common pathogenic fungi. Compounds **6b**, **6e**, **6g**, **6i**, **7b** and **7c** were identified as the most active ones, and the EC_50_ values of these active compounds were further tested. Compared to the commonly used fungicide Azoxystrobin (0.0884 µM), compounds **6b** (0.0544 µM) and **6e** (0.0823 µM) displayed improved activity against *Botrytis cinerea*.

## 1. Introduction

**Figure 1:**
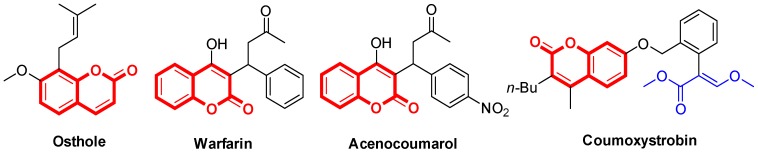
Structures of coumarin-containing drugs and agrochemicals.

Coumarin derivatives are widely distributed throughout nature as secondary metabolites from plants, and the structural modification of coumarin derivatives is a hotspot in the field of natural product chemistry [1,2]. As the structural core, coumarin occurs widely in natural products, drugs and agrochemicals (Figure 1), these important applications have generated considerable interest in this ring system and various fused coumarin derivatives have been reported [3,4,5,6]: Osthole, a natural *O*-methylated coumarin found in *Cnidium Monnieri*, a traditional Chinese herbal medicine that has been used as as a fungicidein China for a long history, and shows antifungal activity against *Rhizoctonia solani* and a broad spectrum of other phytopathogenic fungi [7,8,9]; Warfarin and Acenocoumarol are anticoagulants normally used in the prevention of thrombosis and thromboembolism, function as the vitamin K antagonists [10,11,12]; and Coumoxystrobin (SYP-3375) is a coumarin-containing strobilurin fungicide that contains an (*E*)-methyl 3-methoxy-2-phenylacrylate substructure and displays a broad spectrum of antifungal activity [13,14,15].

**Figure 2:**
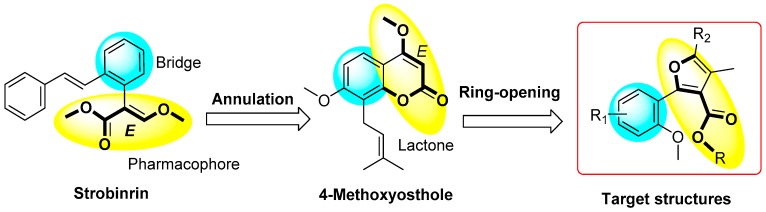
Design strategies of target molecules.

In our previous work, Osthole was used as the lead structure to carry out structural optimization, and some synthesized furan[3,2-*c*]coumarin derivatives showed potent antifungal activity [16,17,18]. In this study, the chemical structure of coumarin can be treated as *O*-hydroxyphenylacrylate lactone, its ring-opening product contains a substructural unit of strobilurin fungicide, the pharmacophore of which is (*E*)-methyl 3-methoxy-2-phenylacrylate (Figure 2). According to our initial design, we synthesized two series of coumarin ring-opening derivatives differentially substituted on the benzene ring, starting from furan[3,2-*c*]coumarin reported as an active lead structure in our previous research [17].

## 2. Results and Discussion

### 2.1. Synthetic Chemistry

**Scheme 1:**
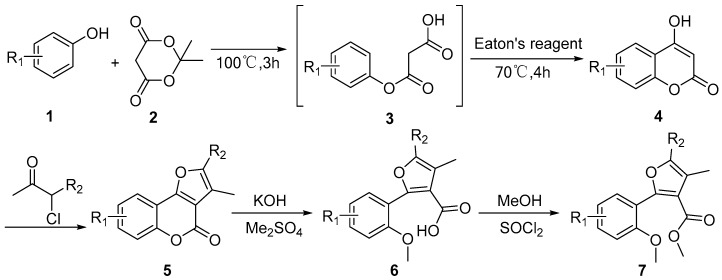
Synthetic routes for target compounds.

Based on the reported synthetic route (Scheme 1) [19],three kinds of substituted phenols **1** and Meldrum’s acid **2** were employed as the starting materials to generate the intermediate maleate **3**, the resulting acetone was removed by rotavapor, then the Eaton’s reagent (phosphoric anhydride + methylsulfonic acid) was added to the residue of the reaction mixture that was continuously stirred at 70 °C for 3 h. Then, the parent structure 4-hydroxycoumarins **4** were prepared in a two-step process, which involved initial transesterification and following oxidative cyclization. Afterward, the reaction of 4-hydroxycoumarin and *α*-haloketone generated furo[3,2-*c*]coumarin derivatives via an efficient tandem *O*-alkylation/cyclisation protocol [17,20]. Then, furan[3,2-*c*]coumarin **5** was used as an intermediate to generate a compound that contains the substructure of the strobilurin fungicide by hydrolysis and methylation [21].Two series of coumarin ring-opening derivatives **6** and **7** were synthesized efficiently in moderate to good yields (from 48% to 75%). The reaction yields were not optimized. The structures of all target compounds **6** and **7** have been confirmed by NMR, IR and HRMS. The yields and structures are also summarized in Chemicals and Methods section.

### 2.2. Antifungal Activity and the Structure-Activity Relationships (SAR)

In general, data presented in Table 1 indicate that the coumarin ring-opening derivatives exhibit certain activities against *Botrytis cinerea*, *Alternaria solani* and *Rhizoctorzia solani* at 50 μM, especially effective to *Botrytis cinerea*, almost half of the synthesized compounds displayed better activity than the positive control Azoxystrobin. Although compound **7c** demonstrated similar activity against *Cucumber anthrax* to Azoxystrobin, most of the target compounds showed rather poor activity against *Gibberella zeae*, *Cucumber anthrax* and *Alternari amali*.

**Figure 3:**
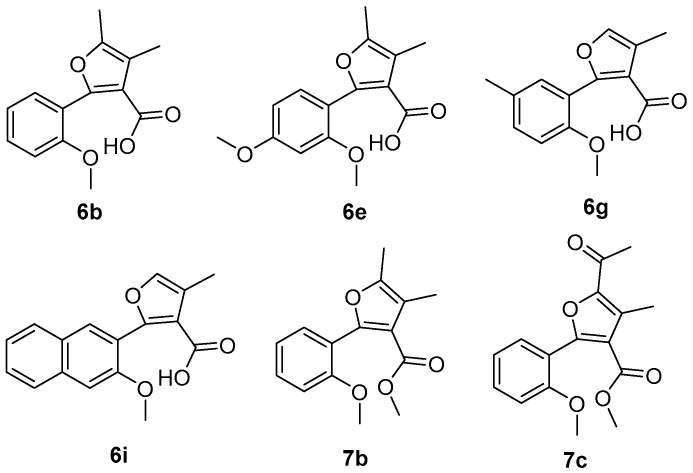
Structures of the most active coumarin ring-opening derivatives.

As eight compounds (**6a**, **6b**, **6e**, **6g**, **6i**, **7b**, **7c** and **7f**) showed relatively effective control against *Botrytis cinerea* and/or *Rhizoctorzia solani*, we further tested the EC_50_ values of these compounds together with Azoxystrobin. As shown in Table 2, we noticed that the EC_50_ values of compounds **6b** and **6e** were as low as 0.0544 and 0.0823 µM against *Botrytis cinerea*, respectively, which proves it is more effective than Azoxystrobin (0.0884 µM). Compound **7b** (0.137 µM) and **7c** (0.182 µM) exhibited equivalent antifungal activity with Azoxystrobin (0.122 µM) against *Rhizoctorzia solani*. Compounds **6b**, **6e**, **6g**, **6i**, **7b** and **7c** were identified as the most active ones among the synthesized coumarin ring-opening derivatives, as shown in Figure 3.

Although the antifungal activity of most of the coumarin ring-opening derivatives has been proven to be rather poor, making it difficult to extract a clear SAR analysis, some broad conclusions still can be drawn from the presented data in Table 1. Firstly, these coumarin ring-opening compounds were noticeably more active against *Botrytis cinerea* than against the five other phytopathogenic fungi. Half of the target compounds displayed better or equivalent activity to the positive control Azoxystrobin. This is highlighted by compounds **6b**, **6e**, **6g** and **6i**. Secondly, the antifungal activity of target compounds varied with the alternation of substituting groups at the benzene ring, as a tentative conclusion, it has a beneficial effect when the R_1_ was H rather than the other substituents, illustrated by the fact that compounds **6a**–**6c**, and **7a**–**7c** generally showed better control against most of the tested fungi than the other compounds.

## 3. Materials and Methods

### 3.1. Chemicals and Methods

All chemicals including substituted phenols, Meldrum’s acid, Eaton’s reagent and 4-Hydroxycoumarin **4a** were purchased from commercial sources (e.g., Crystal Chemicals, Nanjing, China, and Alfa Aesar, Beijing, China) and used without further purification unless otherwise stated. The course of reactions and the purity of products were monitored by TLC using silica gel GF/UV 254. The melting points of these coumarin ring-opening derivatives were determined on an X-4 apparatus (uncorrected), which was bought from Shanghai Tech (Shanghai, China). Nuclear magnetic resonance (^1^H- and ^13^C-NMR) spectra were obtained using a Bruker Avance 400 MHz spectrometer (Bruker Biospin Co., Stuttgart, Germany) in CDCl_3_ solution with TMS as an internal standard. Infrared (IR) spectra were recorded on a Bruker Tensor 27 spectrometer (Bruker Biospin Co.), and samples were prepared as KBr plates. High Resolution Mass Spectrometer (HRMS) spectra were carried out with a ThermoExactive spectrometer (ThermoFisher Scientific Inc., Waltham, MA, USA).

#### 3.1.1. General Procedure for the Synthesis of Compound **4** (Scheme 1)

A mixture of substituted phenol (0.094 g, 1.0 mmol) and Meldrum’s acid (0.144 g, 1.0 mmol) was stirred at 100 °C for 3 h (monitored by TLC), then the small remaining amount of acetone was removed by vacuum. Eaton’s reagent (3 mL) was added to the mixture at 70 °C for 4 h. Then, water was added to this mixture while stirring vigorously. The precipitate was filtered by suction, washed with water, and dried to give a crude product. It was recrystallized from ethanol to afford compounds **4b**–**4d**. (4-Hydroxycoumarin **4a** was obtained from a commercial source).

*4-Hydroxy-7-methoxy-2H-chromen-2-one* (**4b**): white solid, yield 74%, m.p. 264.0–264.5 °C. ^1^H-NMR (300 MHz, DMSO-*d_6_*) δ 12.36 (s, 1H), 7.70 (dd, *J* = 8.4, 0.7 Hz, 1H), 6.96–6.86 (m, 2H), 5.43 (s, 1H), 3.83 (s, 3H).

*4-Hydroxy-6-methyl-2H-chromen-2-one* (**4c**): light yellow solid, yield 54%, m.p. 260.1–261.3 °C; ^1^H-NMR (400 MHz, DMSO-*d_6_*) δ 12.47 (s, 1H), 7.62 (d, *J =* 2.1 Hz, 1H), 7.46 (dd, *J =* 8.4, 2.1 Hz, 1H), 7.27 (d, *J =* 8.5 Hz, 1H), 5.57 (s, 1H), 2.38 (s, 3H).

*4-Hydroxy-2H-benzo[g]chromen-2-one* (**4d**): white solid, yield 51%, m.p. 188.5–186.0 °C; ^1^H-NMR (400 MHz, Acetone-*d_6_*) δ 8.51–8.43 (m, 1H), 8.08–8.01 (m, 1H), 7.94–7.81 (m, 2H), 7.78–7.69 (m, 2H), 5.79 (s, 1H).

#### 3.1.2. General Procedure for the Synthesis of Compound **5** (Scheme 1)

To a stirring solution of substituted 4-hydroxycoumarin **4** (10 mmol) and ammonium acetate (7.7 g, 100 mmol) in toluene (50 mL) and acetic acid (5 mL) was added cholroacetone (5 mL, 62 mmol) or 3-chlorobutan-2-one, and 3-chloropentane-2,4-dione (62 mmol) in a single portion via syringe. The mixture was stirred under reflux for about 10 huntil full conversion of the substrates was indicated by TLC analysis, and then cooled to room temperature and concentrated at reduced pressure. Then 50 mL saturated brine solution was added to the mixture and extracted with EtOAc three times (3 × 50 mL), the extract was washed with 10% NaHCO_3_ solution, organic phase was dried over anhydrous Na_2_SO_4_, and concentrated under reduced pressure. The crude product was purified by silica gel column chromatography using petroleum ether/acetone (20:1 to 5:1) as eluent to give the compound **5** (Table 3).

*3-Methyl-4H-furo[3,2-c]chromen-4-one* (**5a**): white solid, m.p. 155.7–156.3 °C; ^1^H-NMR (400 MHz, Chloroform-*d*) δ 7.87 (dd, *J =* 7.8, 1.6 Hz, 1H), 7.52 (ddd, *J =* 8.6, 7.1, 1.6 Hz, 1H), 7.47–7.40 (m, 2H), 7.35 (td, *J =* 7.5, 1.2 Hz, 1H), 2.39 (d, *J =* 1.4 Hz, 3H); ^13^C-NMR (101 MHz, DMSO-*d_6_*) δ 158.07, 157.30, 152.43, 143.13, 131.35, 125.23, 121.11, 119.81, 117.36, 112.83, 110.42, 8.66. IR (KBr) ν (cm^−1^) 3046, 2927, 1742, 1632, 1581, 1548, 1502, 1445; HR-MS (ESI): *m*/*z* calcd for C_12_H_8_O_3_ ([M + H]^+^) 201.0552, found 201.0546.

*2,3-Dimethyl-4H-furo[3,2-c]chromen-4-one* (**5b**): white solid, m.p. 183.8–184.1 °C; ^1^H-NMR (400 MHz, Chloroform-*d*) δ 7.83 (dd, *J =* 7.8, 1.5 Hz, 1H), 7.5–7.38 (m, 2H), 7.33 (ddd, *J =* 8.2, 7.2, 1.4 Hz, 1H), 2.42 (d, *J =* 1.0 Hz, 3H), 2.30 (d, *J =* 1.1 Hz, 3H); ^13^C-NMR (101 MHz, Chloroform-*d*) δ 158.84, 155.57, 152.22, 150.82, 129.79, 124.24, 120.38, 117.08, 114.06, 113.08, 111.31, 11.51, 8.52; IR (KBr) ν (cm^−1^) 3062, 2956, 1756, 1634, 1619, 1592, 1568, 1439; HR-MS (ESI): *m*/*z* calcd for C_13_H_10_O_3_([M + H]^+^) 215.0708, found 215.0702.

*2-Acetyl-3-methyl-4H-furo[3,2-c]chromen-4-one* (**5c**): light yellow solid, m.p. 178.6–178.9 °C; ^1^H-NMR (400 MHz, Chloroform-*d*) δ 7.99 (dd, *J =* 7.8, 1.5 Hz, 1H), 7.63 (ddt, *J =* 8.5, 7.1, 1.2 Hz, 1H), 7.48 (d, *J =* 8.4 Hz, 1H), 7.45–7.38 (m, 1H), 2.76 (d, *J =* 1.0 Hz, 3H), 2.65 (d, *J =* 0.9 Hz, 3H); ^13^C-NMR (101 MHz, Chloroform-*d*) δ 188.67, 157.76, 157.31, 153.67, 149.14, 132.25, 129.53, 124.80, 121.55, 117.54, 112.13, 111.98, 27.63, 10.23; IR (KBr) ν (cm^−1^) 3028, 2922, 1736, 1674, 1625, 1596, 1545, 1427; HR-MS (ESI): *m*/*z* calcd for C_14_H_10_O_4_ ([M + H]^+^) 243.0657, found 243.0651.

*7-Methoxy-3-methyl-4H-furo[3,2-c]chromen-4-one* (**5d**): light yellow solid, m.p.150.7–151.0 °C; ^1^H-NMR (400 MHz, DMSO-*d_6_*) δ 7.84 (d, *J =* 8.6 Hz, 1H), 7.29 (d, *J =* 2.2 Hz, 1H), 7.17 (dd, *J =* 8.6, 2.2 Hz, 1H), 5.91 (s, 1H), 4.02 (s, 3H), 2.32 (s, 3H); IR (KBr) ν (cm^−1^) 3076, 2920, 1743, 1625, 1600, 1580, 1553, 1446; HR-MS (ESI): *m*/*z* calcd for C_13_H_10_O_4_([M + H]^+^) 231.0657, found 231.0652.

*7-Methoxy-2,3-dimethyl-4H-furo[3,2-c]chromen-4-one* (**5e**): white solid, m.p.143.5–144.0 °C; ^1^H-NMR (400 MHz, Chloroform-*d*) δ 7.72 (d, *J =* 8.5 Hz, 1H), 6.96–6.88 (m, 2H), 3.89 (s, 3H), 2.39 (d, *J =* 1.1 Hz, 3H), 2.28 (d, *J =* 1.1 Hz, 3H); ^13^C-NMR (101 MHz, Chloroform-*d*) δ 161.34, 159.15, 156.31, 153.94, 149.73, 121.33, 113.65, 112.42, 108.96, 106.60, 101.26, 55.68, 11.42, 8.53; IR (KBr) ν (cm^−1^) 3030, 2950, 1748, 1631, 1602, 1511, 1439; HR-MS (ESI): *m*/*z* calcd for C_14_H_12_O_4_ ([M + H]^+^) 245.0814, found 245.0806.

*2-Acetyl-7-methoxy-3-methyl-4H-furo[3,2-c]chromen-4-one* (**5f**): orange solid, m.p. 203.0–204.6 °C; ^1^H-NMR (400 MHz, Chloroform-*d*) δ 7.87 (d, *J =* 8.6 Hz, 1H), 7.01–6.92 (m, 2H), 3.93 (s, 3H), 2.73 (s, 3H), 2.62 (s, 3H); ^13^C-NMR (101 MHz, Chloroform-*d*) δ 188.53, 163.21, 158.07, 158.01, 155.61, 148.60, 129.76, 122.61, 113.21, 109.69, 105.35, 101.45, 55.88, 27.60, 10.28; IR (KBr) ν (cm^−1^) 3088, 2942, 1746, 1672, 1628, 1606, 1551, 1423; HR-MS (ESI): *m*/*z* calcd for C_15_H_12_O_5_([M + H]^+^) 273.0763, found 273.0756.

*3,8-Dimethyl-4H-furo[3,2-c]chromen-4-one* (**5g**): light yellow solid, m.p. 141.7–143.7 °C; ^1^H-NMR (400 MHz, Chloroform-*d*) δ 7.67–7.63 (m, 1H), 7.42 (t, *J =* 1.3 Hz, 1H), 7.34–7.31 (m, 2H), 2.47 (s, 3H), 2.38 (d, *J =* 1.3 Hz, 3H); IR (KBr) ν (cm^−1^) 3064, 2921, 1735, 1632, 1583, 1558, 1501, 1428; HR-MS (ESI): *m*/*z* calcd for C_13_H_10_O_3_ ([M + H]^+^) 215.0708, found 215.0702.

*2-Acetyl-3,8-dimethyl-4H-furo[3,2-c]chromen-4-one* (**5h**): orange solid, m.p. 225.6–226.0 °C; ^1^H-NMR (400 MHz, Chloroform-*d*) δ 7.78–7.75 (m, 1H), 7.42 (dd, *J =* 8.5, 2.0 Hz, 1H), 7.36 (d, *J =* 8.5 Hz, 1H), 2.76 (s, 3H), 2.66 (s, 3H), 2.50 (s, 3H); ^13^C-NMR (101 MHz, Chloroform-*d*) δ 188.66, 157.98, 157.41, 151.91, 149.06, 134.77, 133.32, 129.63, 121.18, 117.27, 111.90, 111.80, 27.64, 20.91, 10.26; IR (KBr) ν (cm^−1^) 3026, 2925, 1733, 1668, 1630, 1593, 1574, 1548, 1506, 1453; HR-MS (ESI): *m*/*z* calcd for C_15_H_12_O_4_ ([M + H]^+^) 257.0814, found 257.0805.

*3-Methyl-4H-benzo[g]furo[3,2-c]chromen-4-one* (**5i**): light yellow solid, m.p. 205.0–205.3 °C; ^1^H-NMR (400 MHz, Chloroform-*d*) δ 8.67–8.59 (m, 1H), 7.94–7.84 (m, 2H), 7.77 (d, *J =* 8.6 Hz, 1H), 7.70–7.61 (m, 2H), 7.46 (q, *J =* 1.4 Hz, 1H), 2.43 (d, *J =* 1.3 Hz, 3H); IR (KBr) ν (cm^−1^) 3028, 2920, 1730, 1617, 1578, 1489; HR-MS (ESI): *m*/*z* calcd for C_16_H_10_O_3_([M + H]^+^) 251.0708, found 251.0702.

*2,3-Dimethyl-4H-benzo[g]furo[3,2-c]chromen-4-one* (**5j**): yellow solid, m.p. 238.5–239.1 °C; ^1^H-NMR (400 MHz, Chloroform-*d*) δ 8.62 (dd, *J =* 8.0, 1.5 Hz, 1H), 7.97–7.81 (m, 2H), 7.75 (d, *J =* 8.6 Hz, 1H), 7.64 (dqd, *J =* 8.4, 6.9, 1.5 Hz, 2H), 2.45 (d, *J =* 1.0 Hz, 3H), 2.35 (d, *J =* 1.0 Hz, 3H); ^13^C-NMR (101 MHz, Chloroform-*d*) δ 158.85, 156.63, 150.72, 148.77, 133.86, 127.98, 127.77, 127.19, 124.53, 123.46, 122.48, 116.99, 114.04, 111.08, 108.35, 11.59, 8.62; IR (KBr) ν (cm^−1^) 2925, 1724, 1632, 1621, 1601, 1593, 1458; HR-MS (ESI): *m*/*z* calcd for C_17_H_12_O_3_ ([M + H]^+^) 265.0865, found 265.0859.

*2-Acetyl-3-methyl-4H-benzo[g]furo[3,2-c]chromen-4-one* (**5k**): yellow solid, m.p. 248.9–249.4 °C; ^1^H-NMR (400 MHz, Chloroform-*d*) δ 8.66–8.55 (m, 1H), 8.00–7.88 (m, 2H), 7.81 (d, *J =* 8.6 Hz, 1H), 7.73–7.66 (m, 2H), 2.78 (s, 3H), 2.67 (s, 3H); ^13^C-NMR (101 MHz, Chloroform-*d*) δ 188.65, 158.20, 157.68, 151.01, 149.04, 134.95, 129.63, 128.96, 128.12, 127.66, 125.09, 123.22, 122.70, 116.98, 111.56, 107.31, 27.64, 10.33; IR (KBr) ν (cm^−1^) 2916, 1737,1673, 1614, 1597, 1556, 1495; HR-MS (ESI): *m*/*z* calcd for C_18_H_12_O_4_ ([M + H]^+^) 293.0814 found 293.0807.

#### 3.1.3. General Procedure for the Synthesis of Target Compounds **6** and **7** (Scheme 1)

A mixture of substituted furocoumarin **5** (10 mmol) and 5% KOH solution (45 mL) was stirred until completely dissolved, then dimethylsulfate (1.0 mL, 10 mmol) was added in a single portion via syringe. The mixture was refluxed for about 10 h until full conversion (monitored by TLC), then cooled to room temperature and acidified to pH = 4–5 with 5% HCl solution, then extracted with EtOAc three times (3 × 50 mL), the extract was dried over anhydrous Na_2_SO_4_, and concentrated under reduced pressure. The crude product was purified by recrystallized from acetone to give the target compound **6**.

Compound **6** (5 mmol) was dissolved in 20 mL CH_3_OH along with SOCl_2_ (30 mmol), the mixture was stirred at room temperature for about 8 h. After completion of the reaction, the mixture was concentrated at reduced pressure, and then 50 mL of water was added, and extracted with EtOAc (3 × 30 mL) and washed with saturated NaHCO_3_ solution (30 mL), washed with water (30 mL), the organic phase was dried over anhydrous Na_2_SO_4_, and concentrated under reduced pressure to afford the target compound **7**.

The reaction yields were not optimized, and the structures of target compounds **6** and **7** are summarized in Table 4.

*2-(2-Methoxyphenyl)-4-methylfuran-3-carboxylic acid* (**6a**): yellow solid, m.p. 134.0–134.2 °C; ^1^H-NMR (400 MHz, Chloroform-*d_3_*) δ 7.43 (ddd, *J =* 15.5, 7.6, 1.7 Hz, 2H), 7.30 (d, *J =* 1.3 Hz, 1H), 7.04 (td, *J =* 7.5, 1.0 Hz, 1H), 6.98 (d, *J =* 8.3 Hz, 1H), 3.82 (s, 3H), 2.24 (s, 3H); IR (KBr) ν (cm^−1^) 3072, 2965, 1680, 1616, 1580, 1557, 1464; HR-MS (ESI): *m*/*z* calcd for C_13_H_12_O_4_ ([M + H]^+^) 233.0814, found 233.0807.

*2-(2-Methoxyphenyl)-4,5-dimethylfuran-3-carboxylic acid* (**6b**): light yellow solid, m.p. 197.0–197.8 °C; ^1^H-NMR (400 MHz, Acetone-*d_6_*) δ 7.40 (td, *J =* 7.4, 1.2 Hz, 2H), 7.07 (d, *J =* 8.5 Hz, 1H), 7.01 (t, *J =* 7.5 Hz, 1H), 3.79 (s, 3H), 2.26 (s, 3H), 2.12 (s, 3H); ^13^C-NMR (101 MHz, DMSO-*d_6_*) δ 165.70, 157.25, 151.44, 147.51, 130.84, 130.70, 120.43, 120.37, 117.49, 115.47, 111.89, 55.72, 11.50, 9.81; IR (KBr) ν (cm^−1^) 3050, 2983, 1683, 1607, 1580, 1565, 1484; HR-MS (ESI): *m*/*z* calcd for C_14_H_14_O_4_([M + H]^+^) 247.0970, found 247.0962.

*5-Acetyl-2-(2-methoxyphenyl)-4-methylfuran-3-carboxylic acid* (**6c**): white solid, m.p. 212.6–213.6 °C; ^1^H-NMR (400 MHz, Acetone-*d_6_*) δ 7.61 (dd, *J =* 7.5, 1.7 Hz, 1H), 7.55–7.47 (m, 1H), 7.19–7.06 (m, 2H), 3.84 (s, 3H), 2.55 (s, 3H), 2.48 (s, 3H); ^13^C-NMR (101 MHz, DMSO-*d_6_*) δ 188.77, 164.83, 157.53, 154.38, 147.53, 132.18, 130.43, 130.13, 120.75, 120.15, 118.96, 112.25, 55.83, 27.76, 10.84; IR (KBr) ν (cm^−1^) 3052, 2937, 1703, 1667, 1606, 1581, 1536, 1494; HR-MS (ESI): *m*/*z* calcd for C_15_H_14_O_5_ ([M + H]^+^) 275.0919, found 275.0912.

*2-(2,4-Dimethoxyphenyl)-4-methylfuran-3-carboxylic acid* (**6d**): light yellow solid, m.p. 140.9–141.2 °C; ^1^H-NMR (400 MHz, Chloroform-*d_3_*) δ 7.38 (d, *J =* 8.4 Hz, 1H), 7.28–7.25 (m, 1H), 6.57 (dd, *J =* 8.5, 2.3 Hz, 1H), 6.53 (d, *J =* 2.3 Hz, 1H), 3.87 (s, 3H), 3.80 (s, 3H), 2.23 (s, 3H); IR (KBr) ν (cm^−1^) 3110, 2962, 1678, 1619, 1597, 1578, 1549, 1481; HR-MS (ESI): *m*/*z* calcd for C_14_H_14_O_5_ ([M + H]^+^) 263.0919, found 263.0911.

*2-(2,4-Dimethoxyphenyl)-4,5-dimethylfuran-3-carboxylic acid* (**6e**): light yellow solid, m.p. 156.9–158.3 °C; ^1^H-NMR (400 MHz, Acetone-*d_6_*) δ 7.31 (d, *J =* 8.4 Hz, 1H), 6.64–6.55 (m, 2H), 3.86 (s, 3H), 3.78 (s, 3H), 2.24 (s, 3H), 2.11 (s, 3H); ^13^C-NMR (101 MHz, DMSO-*d_6_*) δ 165.79, 161.72, 158.57, 151.95, 147.00, 131.63, 116.75, 115.37, 113.18, 105.16, 98.89, 55.80, 55.77, 11.48, 9.89; IR (KBr) ν (cm^−1^) 3076, 2961, 1690, 1615, 1579, 1445; HR-MS (ESI): *m*/*z* calcd for C_15_H_16_O_5_([M + H]^+^) 277.1076, found 277.1068.

*5-Acetyl-2-(2,4-dimethoxyphenyl)-4-methylfuran-3-carboxylic acid* (**6f**): orange solid, m.p. 170.0–171.3 °C; ^1^H-NMR (400 MHz, DMSO-*d_6_*) δ 7.46 (d, *J =* 8.4 Hz, 1H), 6.71–6.63 (m, 2H), 3.84 (s, 3H), 3.76 (s, 3H), 2.45 (s, 3H), 2.43 (s, 3H); ^13^C-NMR (101 MHz, DMSO-*d_6_*) δ 188.62, 165.01, 162.78, 158.92, 154.72, 147.15, 131.37, 130.30, 119.37, 111.65, 105.89, 99.07, 55.95, 55.89, 27.71, 10.88; IR (KBr) ν (cm^−1^) 3071, 2930, 1662, 1614, 1580, 1540, 1450; HR-MS (ESI): *m*/*z* calcd for C_16_H_16_O_6_ ([M + H]^+^) 305.1025, found 305.1018.

*2-(2-Methoxy-5-methylphenyl)-4-methylfuran-3-carboxylic acid* (**6g**): yellow solid, m.p. 143.7–144.2 °C; ^1^H-NMR (400 MHz, Chloroform-*d*) δ 7.29 (d, *J =* 1.2 Hz, 1H), 7.26 (d, *J =* 2.2 Hz, 1H), 7.24–7.19 (m, 1H), 6.87 (d, *J =* 8.4 Hz, 1H), 3.80 (s, 3H), 2.34 (s, 3H), 2.23 (s, 3H); ^13^C-NMR (101 MHz, DMSO-*d_6_*) δ 165.52, 155.27, 154.14, 139.98, 131.38, 131.03, 129.11, 121.41, 120.01, 117.01, 111.90, 55.77, 20.36, 9.93; IR (KBr) ν (cm^−1^) 3078, 2998, 1673, 1552, 1502, 1451; HR-MS (ESI): *m*/*z* calcd for C_14_H_14_O_4_ ([M + H]^+^) 247.0970, found 247.0963.

*2-(2-Methoxy-5-methylphenyl)-4,5-dimethylfuran-3-carboxylic acid* (**6h**): yellow solid, m.p. 213.8–214.5 °C; ^1^H-NMR (400 MHz, Acetone-*d_6_*) δ 7.44–7.39 (m, 1H), 7.34–7.28 (m, 1H), 7.04 (d, *J =* 8.5 Hz, 1H), 3.80 (s, 3H), 2.54 (s, 3H), 2.48 (s, 3H), 2.34 (s, 3H); ^13^C-NMR (101 MHz, DMSO-*d_6_*) δ 188.72, 164.85, 155.52, 154.42, 147.47, 132.47, 130.50, 130.14, 129.56, 120.14, 118.69, 112.20, 55.84, 27.78, 20.37, 10.84; IR (KBr) ν (cm^−1^) 3062, 2997, 1664, 1619, 1577, 1536, 1501, 1456; HR-MS (ESI): *m*/*z* calcd for C_16_H_16_O_5_ ([M + H]^+^) 289.1076, found 289.1069.

*2-(3-Methoxynaphthalen-2-yl)-4-methylfuran-3-carboxylic acid* (**6i**): orange solid, m.p. 137.9–138.6 °C; ^1^H-NMR (300 MHz, DMSO-*d_6_*) δ 8.16–8.08 (m, 1H), 8.00–7.93 (m, 1H), 7.73–7.66 (m, 2H), 7.64–7.55 (m, 2H), 7.46 (d, *J =* 8.5 Hz, 1H), 3.63 (s, 3H), 2.17 (s, 3H); IR (KBr) ν (cm^−1^) 3059, 2987, 1678, 1608, 1594, 1556, 1501, 1471; HR-MS (ESI): *m*/*z* calcd for C_17_H_14_O_4_ ([M + H]^+^) 283.0970, found 283.0963.

*Methyl 2-(2-methoxyphenyl)-4-methylfuran-3-carboxylate* (**7a**): yellow oil, ^1^H-NMR (400 MHz, DMSO-*d_6_*) δ 7.61 (d, *J =* 1.4 Hz, 1H), 7.11 (d, *J =* 8.4 Hz, 1H), 7.03 (td, *J =* 7.5, 1.0 Hz, 2H), 3.73 (s, 3H), 3.61 (s, 3H), 2.11 (s, 3H); IR (KBr) ν (cm^−1^) 2950, 1770, 1713, 1598, 1582, 1552, 1493; HR-MS (ESI): *m*/*z* calcd for C_14_H_14_O_4_ ([M + H]^+^) 247.0970, found 247.0963.

*Methyl 2-(2-methoxyphenyl)-4,5-dimethylfuran-3-carboxylate* (**7b**): yellow solid, m.p. 55.5–56.2 °C; ^1^H-NMR (400 MHz, DMSO-*d_6_*) δ 7.52–7.33 (m, 2H), 7.09 (dd, *J =* 8.4, 1.0 Hz, 1H), 7.01 (td, *J =* 7.5, 1.0 Hz, 1H), 3.72 (s, 3H), 3.59 (s, 3H), 2.25 (s, 3H), 2.04 (s, 3H). ^13^C-NMR (101 MHz, DMSO-*d_6_*) δ 164.78, 156.76, 151.02, 147.86, 130.97, 130.11, 120.54, 119.88, 116.86, 115.34, 111.82, 55.82, 51.48, 11.49, 9.53; IR (KBr) ν (cm^−1^) 3047, 2924, 1710, 1633, 1602, 1581, 1560, 1495; HR-MS (ESI): *m*/*z* calcd for C_15_H_16_O_4_ ([M + H]^+^) 261.1127, found 261.1121.

*Methyl 5-acetyl-2-(2-methoxyphenyl)-4-methylfuran-3-carboxylate* (**7c**): white solid, m.p. 61.4–62.2 °C; ^1^H-NMR (300 MHz, DMSO-*d_6_*) δ 7.58 (dd, *J =* 7.6, 1.7 Hz, 1H), 7.50 (ddd, *J =* 8.4, 7.4, 1.8 Hz, 1H), 7.19–7.05 (m, 2H), 3.74 (s, 3H), 3.64 (s, 3H), 2.45 (s, 3H), 2.43 (s, 3H). ^13^C-NMR (101 MHz, DMSO-*d_6_*) δ 188.78, 163.89, 157.13, 153.99, 147.56, 132.44, 130.13, 129.82, 120.94, 119.25, 118.40, 112.14, 56.00, 52.05, 27.77, 10.62; IR (KBr) ν (cm^−1^) 2948, 1725, 1665, 1610, 1584, 1534, 1492; HR-MS (ESI): *m*/*z* calcd for C_16_H_16_O_5_ ([M + H]^+^) 289.1076, found 289.1068.

*Methyl 2-(2,4-dimethoxyphenyl)-4-methylfuran-3-carboxylate* (**7d**): yellow oil; ^1^H-NMR (400 MHz, DMSO-*d_6_*) δ 7.55 (d, *J =* 1.5 Hz, 1H), 7.31 (d, *J =* 8.4 Hz, 1H), 6.77–6.54 (m, 2H), 3.82 (s, 3H), 3.72 (s, 3H), 3.61 (s, 3H), 2.10 (s, 3H); IR (KBr) ν (cm^−1^) 3052, 2948, 1711, 1618, 1578, 1503, 1455; HR-MS (ESI): *m*/*z* calcd for C_15_H_16_O_5_ ([M + H]^+^) 277.1076, found 277.1069.

*Methyl 2-(2,4-dimethoxyphenyl)-4,5-dimethylfuran-3-carboxylate* (**7e**): yellow solid, m.p. 71.6–73.2 °C; ^1^H-NMR (300 MHz, DMSO-*d_6_*) δ 7.53 (d, *J =* 8.6 Hz, 1H), 6.61 (d, *J =* 2.4 Hz, 1H), 6.56 (dd, *J =* 8.6, 2.4 Hz, 1H), 3.86 (s, 3H), 3.77 (s, 3H), 3.68 (s, 3H) 2.21 (s, 3H), 1.91 (s, 3H). IR (KBr) ν (cm^−1^) 3068, 2922, 1714, 1612, 1582, 1498; HR-MS (ESI): *m*/*z* calcd for C_16_H_18_O_5_ ([M + H]^+^) 291.1232, found 291.1226.

*Methyl 5-acetyl-2-(2,4-dimethoxyphenyl)-4-methylfuran-3-carboxylate* (**7f**): yellow solid, m.p. 120.2–12.4 °C; ^1^H-NMR (400 MHz, DMSO-*d_6_*) δ 7.53 (d, *J =* 9.1 Hz, 1H), 6.69 (dd, *J =* 5.2, 2.3 Hz, 2H), 3.85 (s, 3H), 3.76 (s, 3H), 3.67 (s, 3H), 2.45 (s, 3H), 2.43 (s, 3H). ^13^C-NMR (101 MHz, DMSO-*d_6_*) δ 188.61, 164.08, 162.95, 158.56, 154.37, 147.16, 131.13, 130.01, 118.35, 111.17, 106.06, 99.00, 56.09, 56.00, 51.97, 27.73, 10.66; IR (KBr) ν (cm^−1^) 3020, 2918, 1708, 1662, 1615, 1582, 1439; HR-MS (ESI): *m*/*z* calcd for C_17_H_18_O_6_ ([M + H]^+^) 319.1182, found 319.1175.

*Methyl 2-(2-methoxy-5-methylphenyl)-4-methylfuran-3-carboxylate* (**7g**): yellow oil; ^1^H-NMR (300 MHz, DMSO-*d_6_*) δ 7.57 (q, *J =* 1.2 Hz, 1H), 7.24–7.16 (m, 2H), 6.97 (d, *J =* 8.3 Hz, 1H), 3.67 (s, 3H), 3.59 (s, 3H), 2.26 (s, 3H), 2.08 (s, 3H); IR (KBr) ν (cm^−1^) 2950, 1707, 1617, 1553, 1498, 1438; HR-MS (ESI): *m*/*z* calcd for C_15_H_16_O_4_ ([M + H]^+^) 261.1127, found 261.1122.

*Methyl 2-(2-methoxy-5-methylphenyl)-4,5-dimethylfuran-3-carboxylate* (**7h**): yellow solid, m.p. 117.8–118.1 °C; ^1^H-NMR (400 MHz, DMSO-*d_6_*) δ 7.40 (d, *J =* 2.2 Hz, 1H), 7.32 (dd, *J =* 8.6, 2.3 Hz, 1H), 7.05 (d, *J =* 8.5 Hz, 1H), 3.72 (s, 3H), 3.65 (s, 3H), 2.47 (s, 3H), 2.44 (s, 3H), 2.32 (s, 3H); ^13^C-NMR (101 MHz, DMSO-*d_6_*) δ 188.73, 163.92, 155.12, 154.04, 147.51, 132.76, 130.20, 129.84, 129.79, 119.24, 118.10, 112.05, 56.00, 52.01, 27.80, 20.37, 10.62; IR (KBr) ν (cm^−1^) 3049, 2917, 1708, 1665, 1584, 1505, 1440; HR-MS (ESI): *m*/*z* calcd for C_17_H_18_O_5_ ([M + H]^+^) 303.1232, found 303.1226.

*Methyl 2-(3-methoxynaphthalen-2-yl)-4-methylfuran-3-carboxylate* (**7i**): yellow oil; ^1^H-NMR (300 MHz, DMSO-*d_6_*) δ 8.13 (ddd, *J =* 6.2, 2.3, 1.4 Hz, 1H), 7.97 (dt, *J =* 5.2, 3.1 Hz, 1H), 7.77–7.70 (m, 2H), 7.65–7.57 (m, 2H), 7.50 (d, *J =* 8.5 Hz, 1H), 3.60 (s, 3H), 3.59 (s, 3H), 2.16 (s, 3H). IR (KBr) ν (cm^−1^) 2922, 1727, 1618, 1579, 1493; HR-MS (ESI): *m*/*z* calcd for C_18_H_16_O_4_ ([M + H]^+^) 297.1127, found 297.1120.

### 3.2. Biological Assays

Many experimental protocols for antifungal tests are reported in the literature [22,23].In this study, the antifungal activities of all the synthesized target compounds were carried out at the concentration of 50 μM using mycelia growth inhibitory rate methods, with Azoxystrobin used as the positive control. For the detailed procedure of experimental methods for the antifungal activity, refer to the paper from Department of Plant Pathology, Nanjing Agriculture University [24]. The assay of antifungal activity toward *Botrytis cinerea*, *Alternariasolani*, *Gibberellazeae*, *Rhizoctorziasolani*, *Cucumber anthrax* and *Alternariamali* was carried out on 100 mm × 15 mm Petri plates each contained 10 mL potato dextrose agar, under sterile conditions, on a clean bench in a sterile room. Sterile blank paper disks (0.65 cm in diameter) were placed at a distance 2.5 cm away from the rim of the mycelial colony. The plates were sealed with parafilm, and incubated at 25 °C until mycelial growth had enveloped disks containing the control and had formed crescents of inhibition around disks containing samples with antifungal activity. When the mycelia colony of the control had grown to almost fill the plate, the area of the mycelia colony was measured, and the inhibition of fungal growth in the other plates was determined by calculating the percent reduction in the area of the mycelia colony. The resulting data were collated for each compound, and averages across replicates were used to make a judgment of the overall activity level of the compound.

The antifungal data listed in Table 1 are the screening results of all the compounds against *Botrytis cinerea*, *Alternaria solani*, *Gibberella zeae*, *Rhizoctorzia solani*, *Cucumber anthrax* and *Alternaria mali*, which are the most common phytopathogenic fungi in China.

## 4. Conclusions

In summary, aiming to discover novel Osthole analogs with improved antifungal activity, we have designed and synthesized two series of coumarin ring-opening derivatives through hydrolysis and methylation. Biological testing data showed that some target compounds displayed an altered pattern of biological activity, and compounds **6b**, **6e**, **6g**, **6i**, **7b** and **7c** were identified as the most active ones. The EC_50_ values of these compounds together with Azoxystrobin were further tested. Compared to Azoxystrobin (0.0884 µM), compound **6b** (0.0544 µM) and **6e** (0.0823 µM) displayed improved activity against *Botrytis cinerea*. Further structural optimization of coumarin ring-opening derivatives is well underway, with the aim to improve their levels of antifungal activity.

## Figures and Tables

**Table 1:** Antifungal activity of coumarin ring-opening derivatives (inhibitory rate, %).

Compound	Species ^a^					
	BOT	ALS	GIB	RHI	RHI	ALM
	50 Rate (μM)	50 Rate (μM)	50 Rate (μM)	50 Rate (μM)	50 Rate (μM)	50 Rate (μM)
**6a**	60.3 ^b^	38.0	16.7	31.2	− ^c^	7.8
**6b**	81.0	24.0	28.3	42.2	0	9.8
**6c**	41.3	12.0	0	6.2	0	−
**6d**	49.2	30.0	5.0	23.4	12.1	19.6
**6e**	74.1	7.3	7.1	25.9	18.2	14.8
**6f**	58.6	5.5	0	12.1	0	−
**6g**	77.8	40.0	13.3	34.4	−	17.6
**6h**	33.3	24.0	8.3	20.3	0	−
**6i**	77.6	30.9	26.8	15.5	14.5	38.9
**7a**	51.7	20.0	28.6	37.9	21.8	22.2
**7b**	70.7	23.6	44.6	51.7	14.5	33.3
**7c**	67.2	14.5	30.4	51.7	84.2	25.9
**7d**	25.9	14.5	19.6	34.5	14.5	7.4
**7e**	32.7	0	29.5	50.8	5.8	11.1
**7f**	65.5	20.0	28.6	27.6	38.2	24.1
**7g**	−	9.1	32.1	39.6	14.5	16.7
**7h**	10.3	−	17.8	13.8	0	0
**7i**	0	0	14.3	8.6	0	16.7
**Azoxystrobin**	50.4	31.3	58.2	60.7	89.9	44.5

^a^ BOT, *Botrytis cinerea*; ALS, *Alternariasolani*; GIB, *Gibberellazeae*; RHI, *Rhizoctorziasolani*; CUC, *Cucumber anthrax*; ALM, *Alternariamali*. ^b^ All data are the average value of three replications. ^c^ Test failure or not test.

**Table 2:** EC_50_ determination of some active compounds.

Pathogen	Compound	Toxic Regression	R	EC_50_ (µM)	95% Confidence Interval
BOT	**6a**	Y = 2.4538 + 1.7909x	0.9905	0.1130	0.0966–0.1330
BOT	**6b**	Y = 3.3419 + 1.4690x	0.9994	0.0544	0.0523–0.0566
BOT	**6e**	Y = 2.6307 + 1.7429x	0.9932	0.0823	0.0724–0.0942
BOT	**6g**	Y = 2.1175 + 2.1486x	0.9768	0.0889	0.0696–0.1130
BOT	**6i**	Y = 1.9225 + 2.1902x	0.9779	0.0901	0.0699–0.1160
BOT	**7b**	Y = 2.0301 + 2.0457x	0.9942	0.1090	0.0964–0.1230
BOT	**7c**	Y = 1.7715 + 2.0569x	0.9980	0.1290	0.1200–0.1380
BOT	**7f**	Y = 0.9264 + 2.6215x	0.9954	0.1120	0.1010–0.1250
BOT	**Azoxystrobin**	Y = 3.5635 + 0.9256x	0.9998	0.0884	0.0858–0.0910
RHI	**7b**	Y = 3.3881 + 1.0377x	0.9994	0.1370	0.1320–0.1430
RHI	**7c**	Y = 2.0840 + 1.6967x	0.9950	0.1820	0.1610–0.2050
RHI	**Azoxystrobin**	Y = 3.4242 + 0.9321x	0.9981	0.1220	0.1050–0.1410

The EC_50_ value was the average value of three replications.

**Table 3:** The yields and structures of compound **5**.

Compound	R_1_	R_2_	Yield	Compound	R_1_	R_2_	Yield
5a	H	H	79%	5g	8-Me	H	67%
5b	H	Me	38%	5h	8-Me	Ac	34%
5c	H	Ac	33%	5i	7,8-(CH)_4_	H	55%
5d	7-OMe	H	79%	5j	7,8-(CH)_4_	Me	37%
5e	7-OMe	Me	22%	5k	7,8-(CH)_4_	Ac	45%
5f	7-OMe	Ac	36%				

**Table 4:** The yields and structures of target compounds **6** and **7**.

Compound	R_1_	R_2_	Yield	Compound	R_1_	R_2_	Yield
**6a**	H	H	65%	**7a**	H	H	75%
**6b**	H	Me	55%	**7b**	H	Me	65%
**6c**	H	Ac	55%	**7c**	H	Ac	68%
**6d**	4-OMe	H	60%	**7d**	4-OMe	H	74%
**6e**	4-OMe	Me	56%	**7e**	4-OMe	Me	71%
**6f**	4-OMe	Ac	51%	**7f**	4-OMe	Ac	73%
**6g**	5-Me	H	71%	**7g**	5-Me	H	69%
**6h**	5-Me	Me	50%	**7h**	5-Me	Me	62%
**6i**	4,5-(CH)_4_	H	48%	**7i**	4,5-(CH)_4_	H	58%
